# Medical Expert and Medical Evidence

**Published:** 1875-01

**Authors:** M. A. McClelland

**Affiliations:** Knoxville, Ill.


### Original Communications.

ARTICLE I.

### THE MEDICAL EXPERT AND MEDICAL EVIDENCE.

BY M. A. McCLELLAND, M.D., KNOXVILLE, ILL.

Evidence is that which is submitted to any competent tribunal as a means of ascertaining the truth or falsity of any allegation made before it. It is that which demonstrates or makes clear the truth of the fact or point in issue, either on one side or on the other, and to be competent evidence it must be restricted to the very point in issue. The term "proof," which is commonly used as a synonym for evidence, is employed by legal writers to indicate the effect made upon the mind by the declarations of the witness. "Evidence" they apply to the means used to obtain proof. "Proof is the legal credence which the law gives to any statement; evidence is the legal process by which that proof is made." (Phillips.)

Satisfactory or sufficient evidence is such an amount of proof as would convince the judgment of a common man, and which would lead him to do or act, in matters concerning himself, in the same way as he would if all the facts were within his own personal knowledge.

Competency and admissibility of evidence are questions within the province of the court; sufficiency and effect are matters determined by the jury. A party aggrieved has his redress in a bill of exceptions, and in appeal to a higher court.

One other rule bearing upon evidence is required by courts of justice, viz.: That the *best* evidence the nature of the case will admit of shall be produced, if possible to be had. Strict equity demands that the *best* evidence shall be sought from those best qualified to render *true* testimony. We will see further along how the true spirit of the rule is avoided, especially by those having a bad case to sustain.

The testimony of a mechanic as to the necessity for the removal of a limb, by amputation, could not be received in evidence, for the reason that his avocation in life does not give him the requisite data upon which to found a competent judgment. Here, the separating of things nearly alike, giving to each its due weight, is beyond his skill, therefore he would not be permitted to give evidence in such a case. Such testimony, if given, would not be *competent* evidence.

The testimony of a witness that a limb had received a gun-shot wound, would not relieve the surgeon from paying large damages for malpractice in removing it by amputation, if it was not further shown that the destruction of the vascular and nervous connection with the rest of the body, or some other good reason, rendered the operation necessary. The former evidence, without the latter, would not be *sufficient.*

The testimony of the witness that the prostate gland was not in the head, nor breast, nor stomach, failing to say where it was located, *should* not have been received, because where it *was not* was not the *point* in issue.

It is the first and most important rule of evidence, that the best evidence of which the case is capable shall be presented, for if the best evidence is not offered, it gives rise to the presumption that such evidence would go against the party neglecting to procure it. This is the most important rule in the administration of equal and exact justice, but how frequently we have in its stead the loud assumptions of ignorance and vanity, the records of our courts too frequently show. The reasons for this will be presently shown.

In some of the European states, the courts, in cases of obscure or doubtful physical or mental conditions, summon to their aid physicians, eminent for their knowledge and skill in the several departments of medical science, who, like all witnesses, are allowed, upon the strength of their acknowledged skill, to give opinions in evidence. Such witnesses are called experts.

In general, a witness must depose to facts that are within his own knowledge, but upon questions of science, skill or trade, persons of skill in these avocations may not testify to facts only, but may be permitted to give opinions also, which are received in evidence. "Physicians, when summoned before courts, may appear in a two-fold capacity, that is to say, either as *ordinary* witnesses, to state facts within their own knowledge, or as *skilled* witnesses, to interpret them." (Ordronaux.) It is only in the last capacity they would be designated experts.

From a statement of facts within the personal knowledge of the witness, the jury may draw the deductions and conclusions necessary for the determination of the cause, but upon matters involving abstruse questions in science, trade and commerce, ordinary men, who usually constitute juries, are not acquainted, and consequently are not capable of determining what the facts are in each particular case. It is in the trial of such cases, in which the subject matter is of such a nature that ordinary men have not sufficient knowledge, that the office of the skilled witness is demanded. (*N. E. Glass Co.* v. *Lowell*, 7 Cush. Mass. R. 319.)

To constitute a "medical expert," it requires more than a popular reputation for skill and judgment in things pertaining to disease and its treatment. The *actual* possession of these requirements does not necessarily imply ability to perform the duties of an expert creditably in every case. By this, I would intend very particularly and decidedly to say, that neither great success in the ordinary practice of medicine and surgery, nor popular and well deserved reputation in the practice of the same, constitutes, in any sense, *expertness* in certain branches of medicine and surgery, insanity for instance.

In this disease, the popular idea is, that it is a definite mental condition in every case, capable of clear definition, but the scientific observer recognizes in the mental affection the same characteristics that are met with in physical derangement, namely, a gradual passage from the sane to the insane state, as gradual as health passes into disease. Men of the greatest experience cannot fix the boundary line. What, then, can the ordinary practitioner do, who has perhaps, in a life-long practice, not seen a dozen cases? "Who can say where twilight ends and darkness begins?"

It is a well understood fact by life insurance companies, and, indeed, by intelligent observers generally, that many of the most fatal maladies to which mankind is liable, to the instructed physician are known and determined by certain symptoms and signs, while to unskillful observers they remain *res incognita.* Expertness, therefore, is the result of large observation and experience in special arts and sciences. The field is too large, life is too short, for any one person to accomplish great attainments in all branches of medicine; so where, from the necessity of the case, medical men have to act in the capacity of surgeon, physician and obstetrician, we can scarcely expect to find experts. "However," Dr. Taylor remarks, "medico-legal knowledge does not consist so much in the acquisition of facts, as in the power of arranging them, and in applying the conclusions to which they lead to the purposes of law. A man may be a most skillful surgeon, or a most experienced physician; his mind may be well stored with professional information; yet if he is unable, by the use of simple language, to make his ideas known to others, his knowledge will be of no avail. One far below him in professional standing and experience may make a better medical witness." This should encourage us to improve every opportunity in expressing our ideas in our common tongue as clearly and as connectedly as we may.

We should be stimulated to study by the knowledge, also, "that the mysteries of nature are only capable of solution through the physical sciences. Medicine lacks much of being a positive science. The mechanism of the human body is clear, and that is all. We see the workman and the tools, but the skill that guides the work, and the power that performs it, are as invisible as ever. We have learned nothing but the mechanism of life, and are no nearer its essence. Beyond the mechanical facts, all is mystery in the movements of organization, as profound as the fall of a stone or the formation of a crystal. To the chemist and the microscopist the living body presents the same difficulties, arising from the fact that everything is in a perpetual change in the organism. The fibrine of the blood puzzles the one as much as the globules puzzle the other. In the brain we are sure we do not know how to localize functions; in the spinal cord we think we know something, but there are so many anomalies and seeming contradictions and sources of fallacy, that, beyond the fact of crossed paralysis of sensation, and the conducting agency of the gray substance, I am afraid we retain no cardinal principles discovered since the development of the reflex functions took its place by Sir Charles Bell's great discovery. No satisfactory and conclusive answer has been made to these questions: Why does one cell become nerve and another bone; why one select bile, another fat? How is the solvent agency of the digestive fluid explained? What is the meaning of 'affinity' or 'catalysis' in chemistry? How is it the liver secretes sugar, or how are blood corpuscles formed out of lymph corpuscles, and what becomes of them? What is the office of the vascular glands, the spleen, the thyroid and thymus bodies? What is the function of Peyer's patches? Do these glandules perform the office of lymphatic glands? What theory of animal heat is correct—that of Black, Lavoisier, Crawford, or Liebig, or of no one of them? That of Liebig now being doubted, who will explain the wonder?" (O. W. Holmes, M.D.)

For the want of a little moral courage, medical men now permit themselves to testify foolishly time and again. The simple statement that he is not qualified to testify for want of special study and a practical acquaintance with the case under consideration, is all that is required to release him from the disagreeable task. The idea is too generally prevalent that the medical witness must, under no circumstance, admit his ignorance, lest upon the more practical points of his profession he will have to admit that he is an impostor and a fraud. The fact is patent, and to none more so than the profession, that the profession is made up, to a larger extent than is pleasant to contemplate, of the refuse material of all other professions and occupations in life, and that it is a fraud of the most abominable proportions. This is not so much the fault of the profession, which has among its followers thousands of as good men as there are in the land, as it is of the vicious social and political system under which we live. A profession governed by laws that have no legal character, having as umpire of its claims, to a great extent, only ignorance and self-conceit, how could it be otherwise? In our courts of law, who is the judge of the value of expert testimony? A jury, which is led by its prejudices as frequently as by the testimony; twelve men, who are as often as otherwise the loafers around the court room, men who have never given a thought to these matters, and, as Dr. Ray observes, "nor would have been much better able to draw just conclusions if they had."

It is an acknowledged principle that juries are confined to the investigation and consideration of facts, and to the application of the law as laid down by the court, but if they should have opinions of their own usurping the functions of the witness, or have a law of their own usurping the functions of the judge, what redress? They once might have been impeached. Now, the aggrieved party may move for a new trial with its attendant expenses. Should it happen, as it sometimes does, especially in a case of alleged insanity, that the judge should usurp the function of the expert, what redress? Appeal to a higher court. If the witness usurps the function of judge or jury, the court or counsel promptly administers a rebuke. And this is right. (*Millard* v. *Brown*, 53 N. Y.)

"But when scientific men are called as witnesses, they cannot give their opinions as to the general merits of the cause, but only their opinions upon the facts proved. And if the facts are doubtful, and remain to be found by the jury, it has been held improper to ask an expert who has heard the evidence, what is his opinion upon the case on trial; though he may be asked his opinion upon a similar case, hypothetically stated. Nor is the opinion of a medical man admissible, that a particular act, for which a prisoner is tried, was an act of insanity." (Greenleaf's Evidence.)

Starkie also says, "Such opinions are admissible in evidence, although the professional witness founds them entirely on the facts, circumstances and symptoms, established in evidence by others and without being personally acquainted with the facts."

"Witnesses are *not receivable to state their views* on matters *of legal or moral obligation*, nor on the manner in which *other persons* would probably be influenced, if the parties acted in one way rather than in another. Therefore the opinions of medical practitioners, upon the question, whether a certain physician had honorably and faithfully discharged his duty to his medical brethren, have been rejected."

"But where a libel consisted in imputing to the plaintiff that he acted dishonorably in withdrawing a horse which had been entered for a race, and he proved by a witness that the rules of the Jockey Club, of which he was a member, permitted owners to withdraw their horses before the race was run; it was held that the witness, on cross-examination, might be asked whether such conduct as he had described as lawful, under those rules, would not be regarded by him as dishonorable." (Greenleaf on Evidence, §441, and note.)

So also the medical witness is not to be asked whether the person in question possessed sufficient mind to transact business or make a will, for these are conclusions of law, depending upon the nature of the business, with which the expert is not supposed to know anything. (*Fairfield* v. *Blascomb*, 35 Vt. 398; *White* v. *Bailey*, 10 Mich. 155.

So, also, to the question: "When the defendant has been undeniably subject to fits of epilepsy, should he not have the benefit of every reasonable doubt that might arise as to his sanity?" his humanity would lead him promptly to answer in the affirmative; yet if the opposite party should object, which he probably would do, the question could not be answered. (*State* v. *Klinger*, 46 Mo. 224.)

In a case of epilepsy, when a crime has been committed without any possible motive, without premeditation or passion, openly and in a way not usual with criminals, the expert would have a right to say, that the criminal impulse was most positively the result of the epilepsy. In this case, however, the expert must *know* that the criminal act was immediately preceded by a fit or fits.

The essential requisite in the expert, so far as it relates to his profession, consists in an extensive acquaintance with the principles of Anatomy, Physiology, Therapeutics, Toxicology and Pathology; general, special, medical and surgical. However well prepared the witness may be, he must be particularly careful not "to regard his line of duty as lying in the direction of the success of the one who employed him rather than of the discovery and establishment of truth." The expert is in no sense the advocate of one party or the other, but is sworn to testify to the truth, the whole truth, and nothing but the truth.

Professor Washburn, (Am. Law Review, October, 1866,) in speaking on this subject, says: "That judge or lawyer, however, may consider himself fortunate, who has not, when trying a case involving inquiries to be answered by experts, again and again felt that the opinions advanced by a witness had more to do with promoting the cause of the party in whose favor he appeared than the furtherance of justice. This is partly from the nature of the subjects upon which experts are usually called, and partly from the inability, which many learned and strong-minded men are under, of discriminating in matters of judgment and opinion between what they wish and what they think, or what, upon principles of sound philosophy, they ought to believe."

The expert is brought into the presence of the court and jury in the same way as an ordinary witness, by summoning him to attend by service, personally rendered, of a writ of *subpœna*, which is a judicial paper, commanding the witness to appear before the court having jurisdiction, to testify what he knows in the matter mentioned in the writ. In order to secure the attendance of the witness, the statutes of the different States provide for the reasonable expenses, which are fixed at a certain sum for each day's actual attendance, and for each mile's travel from the residence of the witness to the place of trial and back, without regard to the employment of the witness, or his rank in life.

The sums paid are not alike in all the States, but the principle is believed to be the same. In some States it is sufficient to tender to the witness his fees for travel from his home to the place of trial, and one day's attendance, in order to compel him to appear upon the summons; but in others the tender must include his fees for travel in returning. This is the rule in the courts of the United States. (Conklin's Practice, pp. 265, 266.)

The practice is not uniform in this country, as to the question whether the witness, having appeared, is bound to attend from day to day, till the trial is closed, without the payment of his daily fees; but the better opinion seems to be that without payment of his fees he is not bound to submit to an examination. (Greenleaf on Evidence, § 310.)

"A physician must respond to the *subpœna* of the court, but when put upon the stand as an *expert*, he is not obliged to testify, until he is paid by the party calling him, such a fee as the professional opinion and time spent in court would be worth, under ordinary circumstances, out of court." (*Webb* v. *Page*, 1 Carr. & Kir. 23.) In this case the court said: "There is a distinction between the case of a man who sees a fact and is called to prove it in a court of justice, and that of a man who is selected by a party to give his opinion on a matter with which he is peculiarly conversant from the nature of his employment in life. The former is bound as a matter of public duty to speak of a fact which happens to have fallen within his knowledge; without such testimony the course of justice must be stopped. The latter is under no such obligation; there is no such necessity for his evidence; and the party calling him must pay him." (Redfield on Wills, 2nd Ed., Part 1, 155. Note 46.) In a case in the United States District Court, Mr. Justice Sprague refused to compel the attendance of an interpreter who had neglected to obey a subpœna. The learned Judge said that a similar question had arisen as to experts, and he had declined to issue process to arrest in such cases. When a person has knowledge of any fact pertinent to an issue to be tried, he may be compelled to attend as a witness. In this all stand upon equal ground. But to compel a person to attend merely because he is accomplished in a particular science, art or profession, would subject the same individual to be called upon in every case in which any question in his department of knowledge is to be solved. Thus the most eminent physician might be compelled, merely for the ordinary witness fees, to attend from the remotest part of the district in which a medical question might arise. This is so unreasonable that nothing but necessity can justify it. The case of an interpreter is analogous to that of an expert. It is not necessary to say what the court would do if it appeared that no other interpreter could be obtained by reasonable effort. (*In Case of Roelker*, 1 Sprague's Dec. 276.)

"Once upon the stand, the *skilled* witness is in the position of any professional man consulted in relation to a subject upon which his opinion is sought. His skill and professional experience is his individual capital and property, that he is not obliged to bestow gratuitously upon any person. Neither the public nor private persons have a right to extort his professional services, without a just compensation. On the witness stand, as in his office, his opinions are his own, and he can give them or withhold them as he chooses, for he cannot be compelled to give an opinion, nor be committed for contempt if he refuses to do so. When, however, he has given his opinion, he has now placed it among the *res gestæ* of the evidence, and cannot decline repeating or explaining it on cross-examination. Once uttered to the public ear of the court, it passes among the facts in evidence, and counsel may use it as they please, without any farther compensation to him. The point of declining to give it gratuitously must be made, if at all, at the opening of his examination *in chief*, and will avail him nothing if delayed until the cross-examination." (Ordronaux Jurisprud. of Med., §§ 113, 114.)

A physician who knows nothing as to the cause of death, in cases requiring legal inquests, cannot be forced to testify in respect to opinions in regard to cause of death, without first making a *post mortem* examination, and this he may *refuse* to make, as he may refuse to render any other *personal* service. For such examination it may not always be convenient to pay him in advance; but he has an undoubted right "to demand a legal promise from the party requiring the service, whether the party be a public officer or private citizen." (Ibid., § 116.) "A physician is not entitled to any greater compensation for traveling to and giving evidence at a coroner's inquest, in obedience to a subpœna, than any other witness; but the expenditure of labor and skill by the physician in a post mortem examination will entitle him to additional compensation." (*Gaston* v. *Commissioners of Marion Co., Ind.*, 3 Ind. 497.) Such compensation must be *reasonable.* (*Tuson* v. *Batting*, 3 Esp. N. P. 192.)

I may remark further, for the instruction of the junior members of the profession, that a physician has a right to *refuse* any personal service, especially when no fee is tendered. But if he once enters upon the performance of the service, he becomes responsible for any damage that may accrue through his negligence or want of care. (Edwards on Bailments, p. 98.)

In a case which came before the Court of Exchequer, May, 1868, (*Maxsted* v. *Morris*,) a witness willfully disobeyed a subpœna. In consequence of this, the trial was postponed, at great expense. An arrangement was made by which the witness bound himself to pay a part of the expense. The Chief Baron said : "It must be distinctly understood that in all cases where it appeared to the court that there had been a willful disobedience of a subpœna after proper service, such a contempt of court would be visited with the punishment it deserved." Martin, B. : "It was not to be tolerated that a man should exercise any discretion as to whether he would or would not attend a court in pursuance of a subpœna. Enormous costs were incurred in preparing a case and bringing it down to trial, the whole of which were to be thrown away and wasted, because a man refused to obey a lawful summons to attend as a witness." Pigott, B. : "A subpœna was not to be treated as mere waste paper. Public justice required that persons willfully committing contempt of court should be dealt with in such a manner as to teach them that they could not commit a contempt of court with impunity."

The question may not be one of fees, but of obedience to a simple order to attend and give evidence on matters of *opinion*, irrespective of scientific facts. In *Simpson* v. *Halliday*, 1864, I was required to attend on a subpœna as a skilled witness, to give evidence of *opinion* in reference to the alleged infringement of a patent. The defendant, who summoned me, did not make it in any way a question of fees; but, being wholly unacquainted with the facts of the case, I did not feel in a position at a short notice to appear as a witness for parties of whom I knew nothing. I obeyed the subpœna, as the disobedience of it might have been, in the present uncertain state of the law, a contempt of court; and, after giving my evidence, I requested his Honor to state, for future guidance, whether a skilled witness was compelled to attend under such circumstances as those in which I had attended. I referred him at the same time to the decision of Lord Campbell, in *Betts* v. *Clifford*. The Vice-Chancellor (Wood) said that a court of law never gave an opinion on a speculative question, and there the matter ended.

It would seem, therefore, that a skilled witness, who is not acquainted with *any of the facts* of a case, may be compelled by a subpœna to attend and give evidence on a matter involving scientific opinion alone. Some months before this occurrence, I had given evidence in a similar case, and the defendant, Halliday, seeing that my opinion in that case was favorable to his views, exercised a right to impound my services on his behalf. When some portion of the public press undertake to censure experts for acting as hired witnesses, it may be as well to remember that they may be sometimes unwillingly forced into court by subpœnas which they dare not disobey.

A medical man receiving separate subpœnas to attend trials at different assizes, which are held at or about the same time, the opinion of one of the most learned judges on the bench, was "that in all cases in which there were served separate subpœnas, fixing trials for the same time, the *civil* should give way to the *criminal* case. The former can be postponed; the latter cannot. But if the subpœnas are two criminal cases, the course of a witness should be to attend to that in which the subpœna was first served upon him." (Taylor Princip. and Pract. of Med. Jurisp., vol. 1, 1873, ch. 1.) For the case of *Betts* v. *Clifford*, in which it was held that an expert might disobey a subpœna with impunity, see Taylor's Med.

Jurisp., by Penrose, 1866, p. 38. Professor Ordronaux is inclined to think that the opinion of the court in above case was misapprehended by the reporter.

Witnesses living within the geographical boundaries of a State are considered within the jurisdiction of courts of record ; and in the Federal courts the writ of subpœna cannot be served without the district of the court. By inter-State arrangement depositions can be taken to be used in the trial of causes. If, after being duly summoned, fees paid or tendered or waived, a witness willfully neglects to appear, he is judged guilty of contempt, and, on motion of the party calling him, he is proceeded against by an attachment. An attachment for contempt proceeds not upon the ground of any damages sustained by an individual, but is instituted to vindicate the dignity of the court. The punishment for contempt is fine and imprisonment at the discretion of the court.

The party injured by the non-attendance of a witness has also his remedy, by action on the case for damages at common law; and a further remedy, by action of debt. (Stat. 5 Eliz., ch. 9.)

If a witness resides out of the jurisdiction of the court, his testimony can be obtained only by taking his deposition. This is done in the following manner: The adverse party is notified to be present at the taking, and put interrogatories if he think fit. This notice is served upon him or his attorney, as either may be nearest, allowing time, after the notification, of at least one day for every twenty miles travel, Sundays excluded. Such deposition must be retained by the person authorized to take it, till he shall deliver it with his own hand into the court for which it is taken; or it must, together with a certificate of the causes or reasons for taking it, as above specified, and of the notice, if any, given to the adverse party, be by the magistrate sealed up, directed to the court, and remain under seal till it is opened in court. The usual mode of transmission is by mail, care being taken to inform the clerk, by a proper superscription, of the nature of the document inclosed to his care, for, if opened by him out of court, though by mistake, it will be rejected. (*Beal* v. *Thompson*, 8 Cranch, 70; *Law* v. *Law*, 4 Greenleaf, 167; Greenleaf on Ev., §§ 320, 322.)

The above relates only to civil cases. In criminal cases witnesses are compelled to attend without a tender of fees. But their fees will, in general, be finally paid from the public treasury. In all such cases, the accused is entitled to have compulsory process for obtaining witnesses in his favor. (Greenleaf Ev., § 311.)

"After a witness is sworn and his competency determined, he is first examined by the party calling him. This is the *direct* examination. He is subsequently examined on the same subject by the adverse party. This is the *cross-examination.* In the direct examination, questions that admit of simple affirmative or negative answers are not permitted. Neither must the question assume facts to have been proved which have not been proved, nor answers given that have not been given. The witness is examined on matters of fact within his own knowledge, either of words or actions. In matters of skill and judgment, he may draw inferences and conclusions, which under ordinary circumstances are drawn by the jury alone." (See also *N. E. Glass Co.* v. *Lowell*, 7 Cushing's Mass. R. 319.)

"Cross-examination is one of the principal tests which the law has devised for the ascertainment of truth, and is certainly a most efficacious test. It is by it that the situation of the witness, with respect to parties and the subject of litigation, his interests, motives, inclinations, prejudices, means of obtaining a correct knowledge of the facts to which he bears testimony, the manner in which he has used these means, his power of discovering facts in the first instance, and his capacity for retaining and describing them, are fully investigated and ascertained, and submitted to the consideration of the jury." (Starkie.)

To the foregoing rules of evidence there are some exceptions, as where the witness called appears to be inimical to the party producing him, or unwilling to give evidence, or when an omission in his testimony is evidently caused by want of recollection. However, this matter lies with the discretion of the judge, who will prohibit or permit such questions as he deems best for the course of justice.

Though a witness can testify only to such facts as are within his own knowledge and recollection, yet he is permitted to refresh and assist his memory by the use of *written instruments*, memoranda, or entries in a book. A copy of the original note will generally be objected to. As to the time when the writing thus used to restore the recollection of facts *should have been made*, no precise rule seems to have been established. Such writing should be made at the time the facts were observed, or shortly after. In the Scottish courts, with the exception of memoranda made up at the time, or shortly after the occasion, a witness is not permitted to refer to a written paper. There is one exception to this rule, and this relates to medical or other scientific reports or certificates. Mr. Allison remarks that "the reason for this exception is founded in the consideration that the medical or other scientific facts or appearances, which are the subject of such a report, are generally so minute and detached that they cannot with safety be intrusted to the memory of the witness, but much more reliance may be placed upon a report made out by him at the time when the facts or appearances are fresh in his recollection; while, on the other hand, such witnesses have, generally, no personal interest in the matter, and from their situation and rank in life are much less liable to suspicion than those of an inferior class, or more intimately connected with the transaction in question. Although, therefore, the scientific witness is always called on to read his report, as affording the best evidence of the appearances he was called upon to examine, yet he may be, and generally is, subjected to a farther examination by the prosecutor or a cross-examination on the prisoner's part; and if he is called on to state any facts in the case unconnected with his scientific report, as conversations with persons deceased, confessions heard from the panel, or the like, he stands in the situation of an ordinary witness, and must give his evidence verbally in answer to questions put to him, and can only refer to jottings or memoranda of dates, etc., made up at the time, to refresh his memory like any other persons put into the box." (Allison's Practice, pp. 540, 542.)

The necessity for expert witnesses was recognized in the earlier history of the world. (*Vide* Leviticus, ch. 13.) However, the custom of requiring medical men to appear in courts of law for the purpose of elucidating obscure questions in medicine and surgery, does not go back to very ancient times. In the statutes of Charlemagne it was provided that, in medico-legal inquiries, the judges should avail themselves of the advice and counsel of physicians, who were to be men recognized as eminent in the profession, masters in the art, of high moral character; jurors were to be selected who were *intelligent*, and acquainted with matters pertaining to the case. Legal medicine was, as yet, in its rudimentary state. It belongs to the nineteenth century to refer these questions to a jury whose first qualification must be *ignorance* of the subject matter in dispute.

The great importance of legal medicine was farther recognized (1552) in the constitution published by the Emperor Charles V. Infanticide, wounds, poisoning, abortion, were questions referred by it to medical men. Methods of investigation proper to be employed were indicated, rules for drawing up reports of cases coming into the courts were established. (Renouard's Hist. Med.)

How often, persons especially skilled in medical knowledge have rendered efficient assistance in the furtherance of justice and in protecting the innocent, numberless instances attest. But a few years ago a woman disappeared mysteriously from society. Suspected persons were arrested and examined for her murder; proof being insufficient, they were discharged. Many years after, the remains of an unknown person were exhumed in the vicinity of the supposed murder. Examined by celebrated experts, they were found to be those of the woman who had disappeared. This led to the second arrest of the supposed criminals, their conviction and punishment.

In the case of a person charged with murdering a child by piercing its head with an awl, Mr. Seldon, a noted surgeon, was able to demonstrate to the satisfaction of the jury, that the hole in the skull which was said to have been made with the awl, was a natural opening for the passage of a vein; thereupon the accused was honorably discharged.

The application of these principles of the science of medicine rightly belongs to the medical practitioner. When he was appointed especially an "expert," by the State, it was an unimportant matter whether the general practitioner gave any thought or not to questions that might acquire a legal character. Then, given in charge the duty of examining into the nature of the case, and making a detailed report thereon, he was enabled to discharge the duty under the most favorable circumstances. He made his examination of the dead body or other subject, submitted to him, at his leisure; consulted his authorities on disputed points; weighed doubtful opinions in his mind till they were entirely clear; then drew his conclusions, and formed his opinions, at the same time arranging in regular and available combinations all the reasons therefor. In this way and under these circumstances, great honor was reflected upon himself and his profession.

In this country there is no forensic physician appointed and paid by the State, to perform the important duties of medico-legal examinations. Instead of there being a forensic physician, a forensic surgeon and a forensic obstetrician, the examination of all questions of a medical or surgical character devolves upon one individual, who is expected to be informed upon all branches of medical knowledge. Nor is he given any time for preparation, either in looking up the facts bearing upon the case or for acquiring any knowledge of the legal aspect of the case upon which he is required to give testimony. And yet we are told that "the opinions of experts are not so highly regarded now as they formerly were." Judge Davis, of the Supreme Court of Maine, says, (1 Redfield on Wills, ch. iii, § 13,) "If there is any kind of testimony that is not only of no value, but even worse than that, it is, in my judgment, that of medical experts. They may be able to state the diagnosis of a disease more learnedly; but upon the question, whether it had at a given time reached such a stage that the subject of it was incapable of making a contract, or irresponsible for his acts, the opinion of his neighbors, if men of common sense, would be worth more than that of all the experts in the country." This is stating the matter very strongly, but it must be admitted that to a great extent it is true. However, the learned judge might have qualified his statement, so that it would not have been so sweeping in its application. This, Judge Redfield, when commenting on the same case, does. He says: "We are more and more confirmed in an opinion that the difficulty comes largely from the manner in which the witnesses are selected. If the State or the courts do not esteem the matter of sufficient importance to justify the appointment of public officers, * * * it is certain the parties must employ their own agents to do it; and it is perhaps almost equally certain, that if it be done in this mode it will produce two trained bands of witnesses, in battle array against each other, since neither party is bound to produce, or will be likely to produce, those of their witnesses who will not confirm their views." (W. & S. Med. Juris., b. 1, § 294.)

In the above case we think Judge Davis erred in assuming that the expert could be questioned as to a conclusion in law, whereas that is a function peculiar to the court. The expert cannot be judge and witness any more than he can be expert and jury. In such a case, or one in which insanity is the question under consideration, the expert may be required to give his opinion as to the state of mind, but not as to the responsibility of the agent.

(To be continued.)

